# The transfer accuracy of digital and conventional full-arch impressions influenced by fixed orthodontic appliances: a reference aid–based in vitro study

**DOI:** 10.1007/s00784-022-04721-5

**Published:** 2022-09-15

**Authors:** Maximiliane Amelie Schlenz, Katharina Klaus, Alexander Schmidt, Bernd Wöstmann, Marco Mersmann, Sabine Ruf, Niko Christian Bock

**Affiliations:** 1 Department of Prosthodontics, Dental Clinic of the Justus Liebig University Giessen, Schlangenzahl 14, 35392 Giessen, Germany; 2 Department of Orthodontics, Dental Clinic of the Justus Liebig University Giessen, Schlangenzahl 14, 35392 Giessen, Germany

**Keywords:** Intraoral scanner, Digital dentistry, Accuracy, Precision, Trueness, Full-arch impression, Fixed orthodontic appliance

## Abstract

**Objectives:**

The aim of this in vitro study was to investigate the influence of fixed orthodontic appliances (FOAs) on the transfer accuracy of full-arch impressions by five intraoral scanners (IOSs): CS3600, Primescan, Trios 4, Medit i500, Emerald S, and one conventional alginate impression (CAI).

**Materials and methods:**

To compare the data with the actual model situation, an established reference aid–based method was applied. A test model with human teeth was used and modified for each testing group, resulting in five different settings: natural teeth (group A), metal brackets without/with wire (groups B/C), ceramic brackets without/with wire (groups D/E). A total of 300 (*n* = 12 × 5 × 5) scan datasets of IOSs were analyzed using a 3D software (GOM Inspect) and 60 (*n* = 12 × 5) plaster casts of CAI were measured with a coordinate measurement machine. The deviations between the reference aid and the impressions were determined.

**Results:**

For all groups with brackets (B to E), IOSs showed a higher transfer accuracy compared to CAI, even for long-span distances. However, some significant differences between the IOSs were observed (*p* < 0.05).

**Conclusions:**

Within the limitations of this in vitro study, IOSs can be recommended for impressions with and without FOAs, even if CAI showed the smallest average deviations in settings without FOAs.

**Clinical relevance:**

IOSs are widely used in orthodontics and the current study demonstrated that their use enables fast impression taking even in settings with fixed orthodontic appliances. In addition, for these settings, the transfer accuracy is higher than with conventional alginate impressions. Nevertheless, a re-investigation in a clinical setting should be performed to verify the current in vitro findings.

**Supplementary Information:**

The online version contains supplementary material available at 10.1007/s00784-022-04721-5.

## Introduction

Orthodontic treatment involves the use of study casts at multiple time points and for a variety of purposes. The use of conventional alginate impressions (CAIs) and the subsequent manufacturing of plaster casts have been the gold standard for decades and show sufficient accuracy. However, fixed orthodontic appliances (FOAs) on tooth surfaces, e.g., buccal brackets, cause several undercuts, which might lead to deformations or tear-out effects of the alginate impression material, resulting in an incorrect display of the intraoral situation, as it is described for conventional impression taking with subsequent plaster casting in the literature for aged dentitions with several undercuts [1].

Meanwhile, IOSs are commonly used in orthodontics for pre-treatment diagnostics or manufacturing of orthodontic appliances [2–9]. Several studies showed a higher accuracy for digital impression taking compared to CAI and described additional advantage like reduced garbage, higher patient comfort, and no storage space of plaster casts [10–13]. However, intraoral scanning of teeth with FOAs is completely different from intraoral scanning of “smooth natural teeth.” In the current literature, contradictory findings regarding the influence of buccal brackets on the accuracy of full-arch impressions are described. Two in vitro studies reported no clinically relevant effect of buccal brackets and wires on the impression accuracy [14, 15], whereas another in vitro study using artificial saliva as well as a clinical trial described a significant reduction of accuracy due to the presence of attachments [16, 17]. Contradictory data are also described regarding the influence of bracket material—metal or ceramic. While metal attachments have been found to cause more disturbances than ceramic attachments in one investigation [16], another investigation reported opposing data [18]. General difficulties have been described when brackets were supposed to be captured in detail [19]. An in vivo trial using metal attachments and digital superimpositions of the resulting scans determined minor deficits around the attachments, particularly in posterior areas; those, however, were considered clinically irrelevant [20].

A possible reason for this obscure data situation might be the investigation method. Previous studies describing an assessment of “accuracy” only superimposed two models or datasets using a best-fit algorithm of 3D software. While this approach at the best describes the discrepancy between the two investigated models or datasets [18, 21], no information on the accuracy of the impression technique itself can be drawn due to the lack of a proper reference. The problem with best-fit algorithms is that errors are compensated across the complete jaw, as the best-fit algorithm calculates as a method of approximation minimizing mesh errors [21, 22]. A reference is mandatory for the analysis of transfer accuracy in terms of trueness and precision of the method [23–26]. Thus, the existing limited amount of even partially contradictory data requires a sound investigation under standardized conditions using a proper reference.

As several studies describe a higher patient acceptance of digital impressions carried out by IOSs compared to conventional impression techniques [10–13], benefits regarding the required time for the different methods have been assessed differently [11, 13, 18, 27]. In young orthodontic patients without any FOA, the required chair-side time for both procedures was found to be equal [11, 12] respectively significantly shorter for alginate impressions, whereby the following laboratory time resulted in comparable summed-up times for IOSs and CAIs [27]. A current in vitro study assessed the time needed for IOS of plaster casts with different FOAs of variable materials compared to models without any FOA and found the scanning time to be shortest in models without any FOA, followed by models with metal brackets and finally models with ceramic brackets [18].

Overall, reliable information regarding the transfer accuracy in terms of trueness and precision of digital impressions as well as the time efficiency of IOSs compared to the current gold standard CAI in orthodontic patients with FOAs are scarce. Therefore, the aim of this in vitro study was to systematically investigate the influence of different buccal orthodontic brackets without/with wire on the transfer accuracy of full-arch impressions by using an established reference aid–based method [24].

The first null hypothesis was that no significant differences can be found between the five IOSs and CAIs regarding the transfer accuracy between natural teeth and FOAs. The second null hypothesis was that no significant differences exist between the five IOSs and CAIs in terms of the amount of time required for impression taking and further processing under natural conditions as well as with FOAs.

## Materials and methods

### Test model

**Fig. 1:**
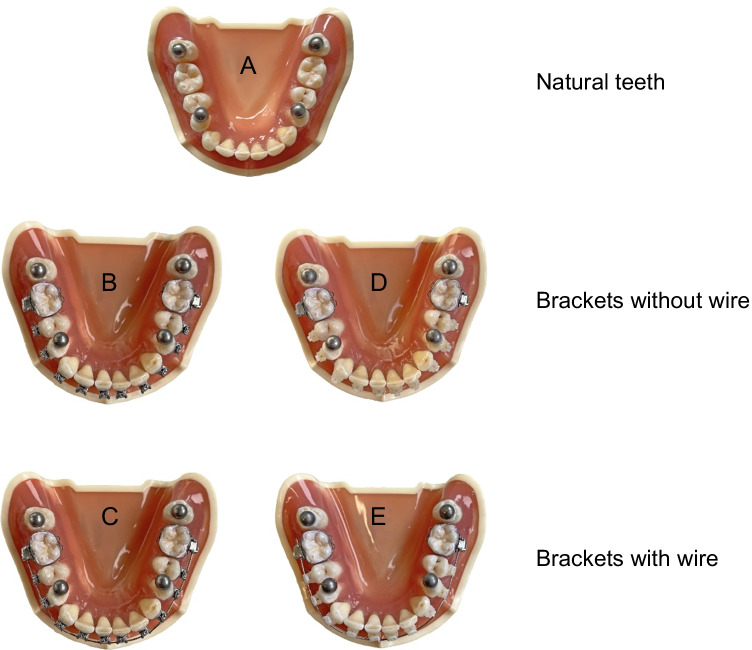
Top view of the test model with natural teeth (**A**), with metal brackets without wire (**B**), metal brackets with wire (**C**), ceramic brackets without wire (**D**), and ceramic brackets with wire (**E**), where the four high-precision bearing spheres have been bonded

To simulate a clinical close setup of a permanent dentition including second molars, a test model of the mandibular jaw (ANA-4, frasaco GmbH, Tettnang, Germany) was modified with natural human teeth embedded in pink-colored acrylic resin (PalaXPress Kulzer, Hanau, Germany; Fig. 1). Therefore, caries-free, undamaged human teeth, which had to be extracted for therapeutic reasons due to periodontal breakdown, were used. Patients and dentists obtained informed consent, and approval of the ethics committee of the medical faculty at the Justus Liebig University Giessen, Germany (Ref. no. 143/09), was given. All methods were performed in accordance with the relevant guidelines and regulations.

The test model was used to simulate the following orthodontic settings:

- Group A: Natural teeth
- Group B: Metal brackets without wire
- Group C: Metal brackets with wire
- Group D: Ceramic brackets without wire
- Group E: Ceramic brackets with wire.

For groups B to E, buccal orthodontic brackets were directly bonded to human teeth 5–5 using light-curing adhesive, and metal bands were cemented on both first molars applying a Glass Ionomer Luting Cement (Fig. 1). All orthodontic materials used for the present study are listed in Table 1.

**Table 1:** Orthodontic materials used in this study

Material	Product name	Manufacturer
Bands	Unitek Victory Series First Molar Bands	3 M, St. Paul, MN, USA
Metal brackets	Tip-Edge PLUS Stainless Steel Brackets	TP Orthodontics Inc., La Porte, IN, USA
Ceramic brackets	Tip-Edge PLUS Aesthetic Brackets	TP Orthodontics Inc., La Porte, IN, USA
Wire	TP Original Wire Coil Premier Plus 0.46 mm .018ʺ	TP Orthodontics Inc., La Porte, IN, USA
Ligatures	Select-Tie Ligature Ties “Metallic Silver” for metal brackets and “Pearl” for ceramic brackets	G&H Orthodontics, Franklin, IN, USA
Cement	Ketac Cem radiopaque Glass Ionomer Luting Cement	3 M, St. Paul, MN, USA
Adhesive	Transbond LR Adhesive	3 M, St. Paul, MN, USA
Etchant and primer	Transbond Plus Self-Etching Primer	3 M, St. Paul, MN, USA

An experienced orthodontist (N.C.B.) inserted the orthodontic fixed appliance according to an established technique [28]. For groups C and E, the wire was passively inserted into the rectangular tube of the molar bands and attached to the brackets using elastomeric ligature ties (Table 1).

### Reference aid and reference dataset

**Fig. 2:**
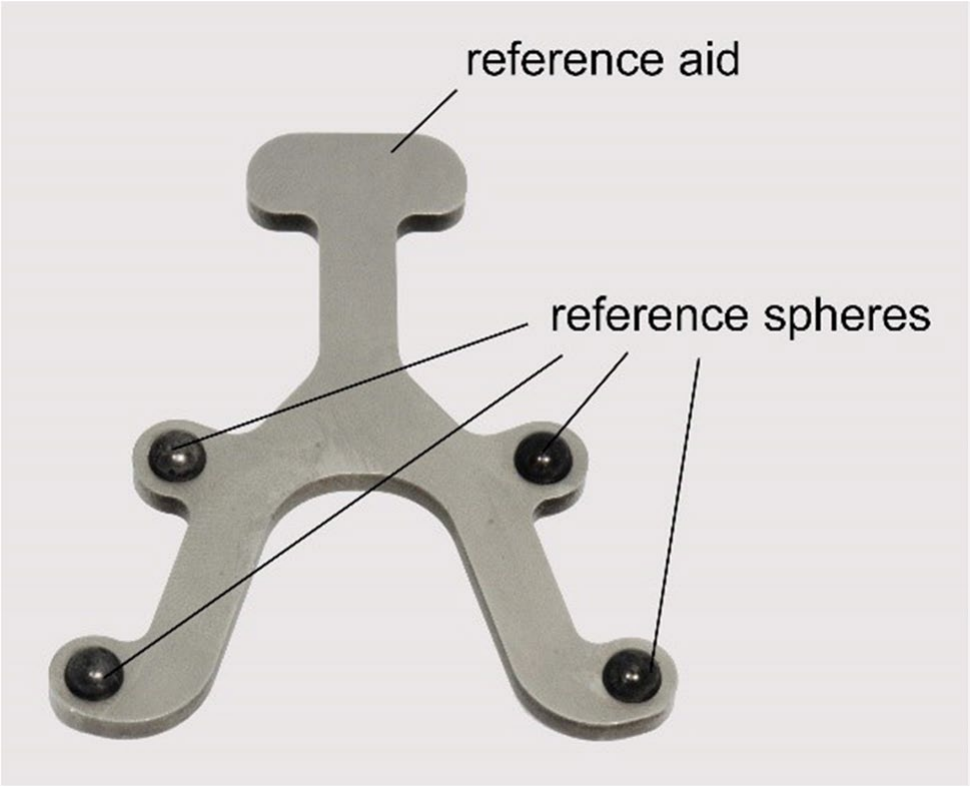
Reference aid with the four reference spheres inserted

According to previous studies investigating the transfer accuracy of full-arch impression taking [24–26], a reference aid was used to position four high-precision bearing spheres (1.3505 100Cr6 DIN5401 [29], TIS GmbH, Gauting, Germany) with a roundness of 5000 ± 5.63 μm [30] on the occlusal surface of the mandibular jaw (Fig. 2). The spheres were reversibly bonded with flowable composite (Grandio Flow, Voco, Cuxhaven, Germany). This method allows a reproducible placement of reference spheres with a precision of less than 10 μm [26].

To create the reference dataset, the reference aid with the four spheres inserted was measured ten times using a coordinate measurement machine (CMM, Thome Präzision GmbH, Messel, Germany) with the corresponding 3D software (X4 V10 GA × 64, Metrologic Group, Meylan, France). Afterwards, the mean value of each sphere position was determined and the reference dataset was stored in an initial graphics exchange specification (IGES) format.

### Impression taking

Before impression taking, the test model was mounted in a phantom head (P-6/5 HGB, frasaco GmbH) and the IOSs were calibrated according to manufacturer’s instruction [31]. Each group (A to E) was investigated in the same sequence, beginning with digital impression taking of CS 3600 (“CAR,” version 7.0.23, Carestream Dental LLC, Atlanta, GA, USA), followed by Primescan (“PRI,” version 5.1.3, Dentsply Sirona, Bensheim, Germany), Trios 4 POD wireless (“TIO,” version 20.1.3, 3Shape, Copenhagen, Denmark), Medit i500 (“MED,” version 2.3.6, Medit, Seoul, South Korea), and Planmeca Emerald S (“EME,” version 6.0.1.812, Planmeca, Helsinki, Finland). To ensure comparable testing conditions, the same scanning path was applied—starting on the occlusal surfaces, going on to the oral surfaces and returning on the buccal surfaces [32]. Subsequently, scan datasets were exported from the IOSs in a standard tessellation language (STL) format.

After digital impression taking, one conventional impression was taken using alginate (“CAI,” Cavex Orthotrace, batch no. 210204, Cavex Holland, Haarlem, Netherlands) standardly mixed in a Migma 200 (Mikrona Technologie, Schlieren, Switzerland) according to the manufacturer’s instruction with a full-arch metal tray (Ehricke Stainless Steel, Orbis Dental, Germany). After 10 min while the alginate impression was stored moist, the CAI was casted with type IV dental stone (Fujirock EP, batch no. 2102183, GC Europe, Leuven, Belgium) [33]. The whole impression taking procedure was repeated twelve times, meaning that for each group (A to E) a total of 60 (12 × 5) IOS datasets were acquired and twelve plaster casts were manufactured.

The plaster casts were stored under laboratory conditions for a minimum of 5 days before measurement. Digital and conventional impression taking as well as measurements was performed by one experienced operator (M.M.) under laboratory conditions with a room temperature of 23 ± 1 °C and a humidity of 50 ± 10%. The required time for digital and conventional impression taking as well as conventional plaster model casting (CPC) was measured with an electronic time clock. For CPC, the 10 min time between CAI and plaster casting as well as the plaster hardening time was not included.

### Analysis of transfer accuracy

**Fig. 3:**
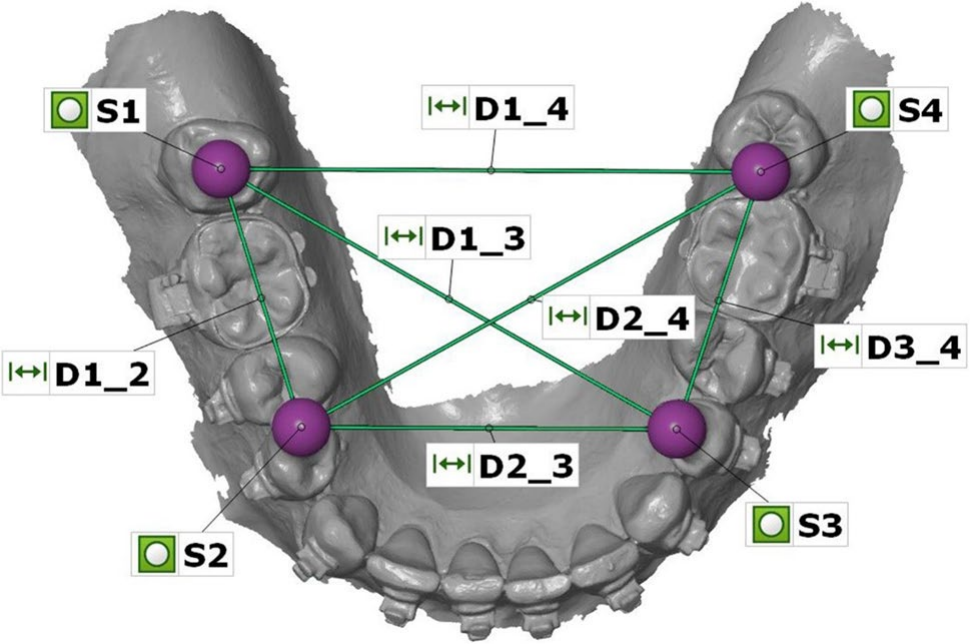
Measurement of linear distances (D1_2, D1_3, D1_4, D2_3, D2_4, D3_4) between the centers of the four spheres (S1-S4) displayed in GOM Inspect analysis software

The STL datasets of the IOSs were imported in the 3D analysis software GOM Inspect (version 2020, GOM GmbH, Braunschweig, Germany) as *actual data* and the reference dataset of the reference aid as *CAD data*. Because actual datasets of IOSs were imported as linked point clouds, the first four spheres were constructed in the position of the spheres using fitting elements (Gauß best fit, 3 Sigma). Afterwards, the linear distances (D1_2, D1_3, D1_4, D2_3, D2_4, D3_4) between the four spheres (S1-S4) were measured for each IOS dataset (Fig. 3). Finally, deviations between reference dataset and the IOS dataset were calculated.

For analysis of the CAI, the plaster casts were measured ten times with the CMM using a specifically programmed mode of operation which automatically probed the individual spheres, and allowed to calculate the distances between the centers of the individual spheres. Subsequently, the mean values of each sphere were calculated and further measurements of linear distances were accomplished with the corresponding 3D software.

The statistical analysis was performed using SPSS Statistics (version 26, IBM, Armonk, NY, USA). The data was subjected to a two- respectively three-way ANOVA. Due to the heterogeneity of variances, the SPSS procedure GENLINMIXED was applied [34]. For a detailed evaluation of the different groups and distances, one-way ANOVAs with impression technique as a six-step factor were performed. The analyses were carried out as a non-parametric Kruskal–Wallis test due to extreme outliers. The level of significance was *p* < 0.05.

Accuracy in terms of trueness and precision is given according to ISO 5725–1 [35]. Meaning that, for trueness, the mean deviations between the impressions and the reference aid were described and for precision the standard deviations were used.

As no manual measurements were performed and only one investigator was involved, no intra- or interrater reliability was determined.

## Results

**Fig. 4:**
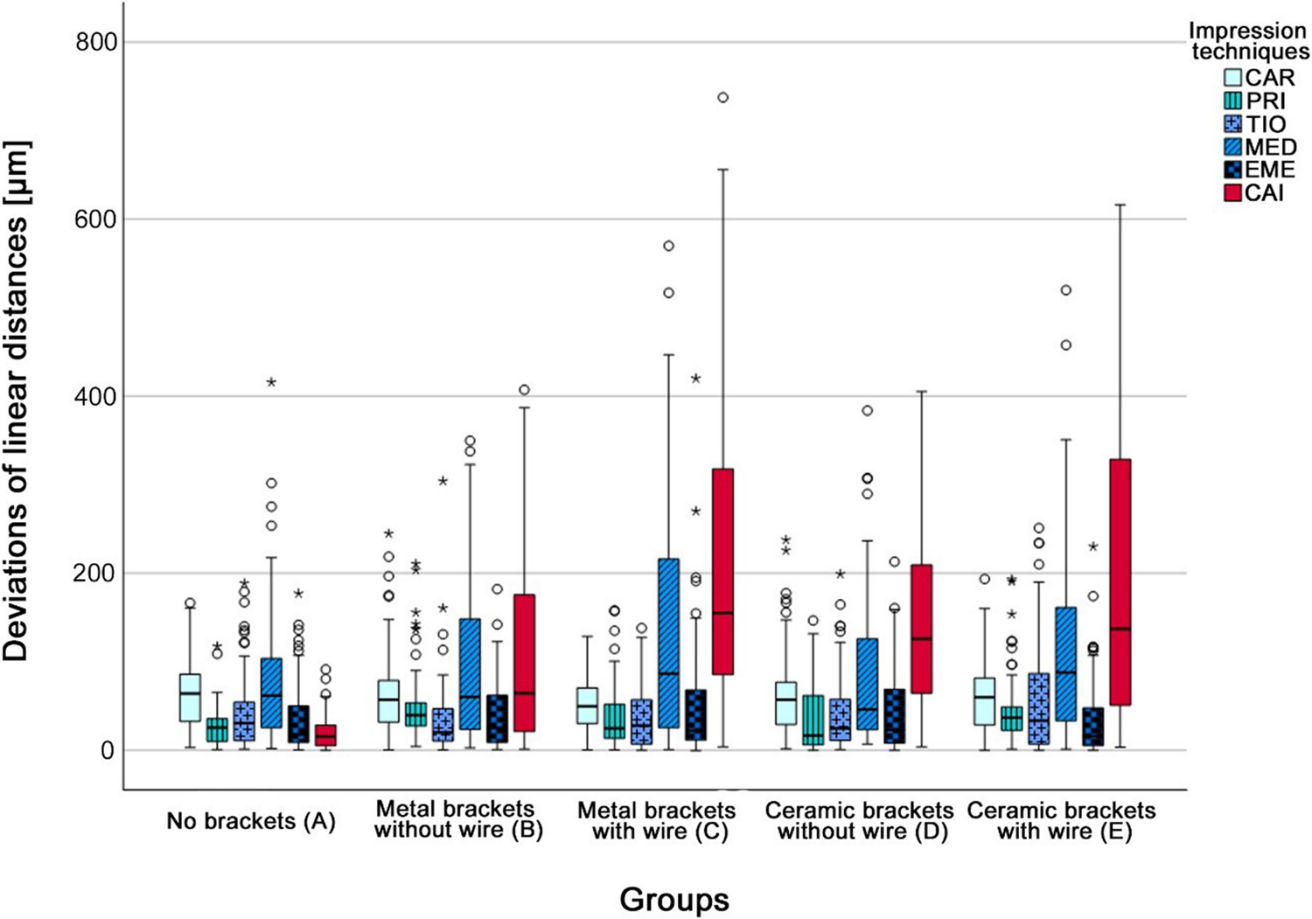
Boxplot diagrams showing the pooled data of the deviations of the linear distances (D1_2, D1_3, D1_4, D2_3, D2_4, D3_4) for the different impression techniques (CAR = CS 3600, PRI = Primescan, TIO = TRIOS 4, MED = Medit i500, EME = Emerald S, CAI = conventional alginate impression) in groups A–E; outliners (Ο), extreme values (*)

**Fig. 5:**
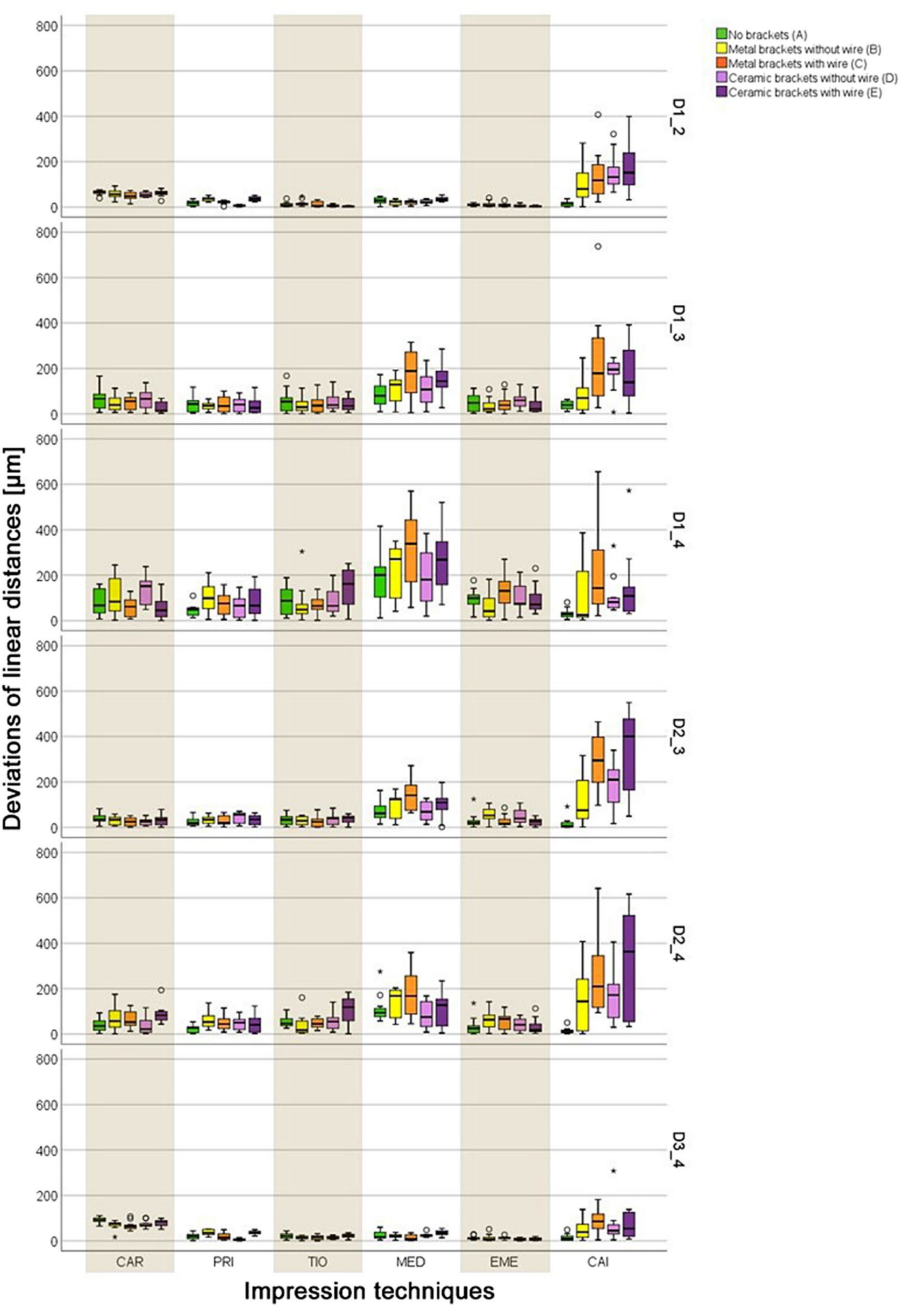
Boxplot diagrams showing the deviations of the linear distances (D1_2, D1_3, D1_4, D2_3, D2_4, D3_4) in groups A–E for the different impression techniques (CAR = CS 3600, PRI = Primescan, TIO = TRIOS 4, MED = Medit i500, EME = Emerald S, CAI = conventional alginate impression); outliners (Ο), extreme values (*)

The calculated pooled deviations between the reference dataset and the data of the six impression techniques for the linear distances (D1_2, D1_3, D1_4, D2_3, D2_4, D3_4) separated into the five groups (A to E) are displayed in Fig. 4 and Table 2. All IOSs showed lower deviations (range: 28 ± 23 to 141 ± 140 µm) compared to the CAI (range: 21 ± 20 to 212 ± 204 µm), except for group A, where CAI showed the lowest deviation (21 ± 20 µm). For all groups A–E, statistically significant differences (*p* < 0.001) existed between the six impression techniques (Table 2). When evaluating trueness and precision of each separate impression technique, MED exhibited the largest deviations in comparison to all other IOSs, but still showed lower values than CAI (Table 2). CAI in settings with FOAs showed high deviations. Some IOSs were more affected by FOAs than others and also the bracket material (metal or ceramic) as well as the presence of a wire seems to have an impact on the transfer accuracy (Table 2). The detailed data for each separate linear distance (D1_2, D1_3, D1_4, D2_3, D2_4, D3_4) and impression technique are displayed in Fig. 5 as well as Supplementary Information (Tables 1 and 2), which include the respective *p* values.

**Table 2:** Pooled data of the linear distances (D1_2, D1_3, D1_4, D2_3, D2_4, D3_4) for the different groups A–E reported in ascending order with respect to the mean value of the different impression techniques (CAR = CS 3600, PRI = Primescan, TIO = Trios 4, MED = Medit i500, EME = Emerald S, CAI = conventional alginate impression) as mean for trueness [µm] and standard deviation (SD) for precision [µm] according to the International Organization for Standardization (ISO) 5725–1^35^. In addition, the respective *p* values of the comparison within each group (A–E) as well as the comparison within each impression technique are given

Group	Mean (trueness) [µm] ± SD (precision) [µm]						*p* value
	PRI	TIO	EME	CAR	MED	CAI	
No brackets (A)	28 ± 23	44 ± 45	38 ± 42	64 ± 38	84 ± 79	21 ± 20	< 0.001
Metal brackets without wire (B)	51 ± 42	34 ± 44	40 ± 39	64 ± 49	99 ± 92	103 ± 103	< 0.001
Metal brackets with wire (C)	39 ± 36	36 ± 33	47 ± 55	52 ± 31	141 ± 140	202 ± 163	< 0.001
Ceramic brackets without wire (D)	34 ± 34	41 ± 42	43 ± 45	65 ± 50	84 ± 83	143 ± 95	< 0.001
Ceramic brackets with wire (E)	45 ± 40	59 ± 67	33 ± 43	57 ± 36	117 ± 108	212 ± 204	< 0.001
Mean (A–E)	39 ± 36	43 ± 48	59 ± 84	60 ± 42	105 ± 105	136 ± 149	
*p* value	< 0.001	0.001	< 0.001	0.050	< 0.001	< 0.001

Generally, the smallest deviations were determined for the two short distances (D1_2, D3_4) and the largest deviations for the longer intermolar distance (D1_4). Within the IOSs, MED exhibited the largest deviations for trueness and precision (except for D1_2 and D3_4), and the two groups with brackets and wire (C and E) displayed higher deviations than the two groups with brackets only (B and D). The latter was also seen for TIO and CAI. For EME, CAR, and PRI, some groups showed larger and others smaller deviations in settings with respectively without wire (Fig. 5).

Therefore, the primary null hypothesis, that no significant differences exist between the five IOSs and CAIs regarding the transfer accuracy between natural teeth and FOAs, has to be rejected.

**Fig. 6:**
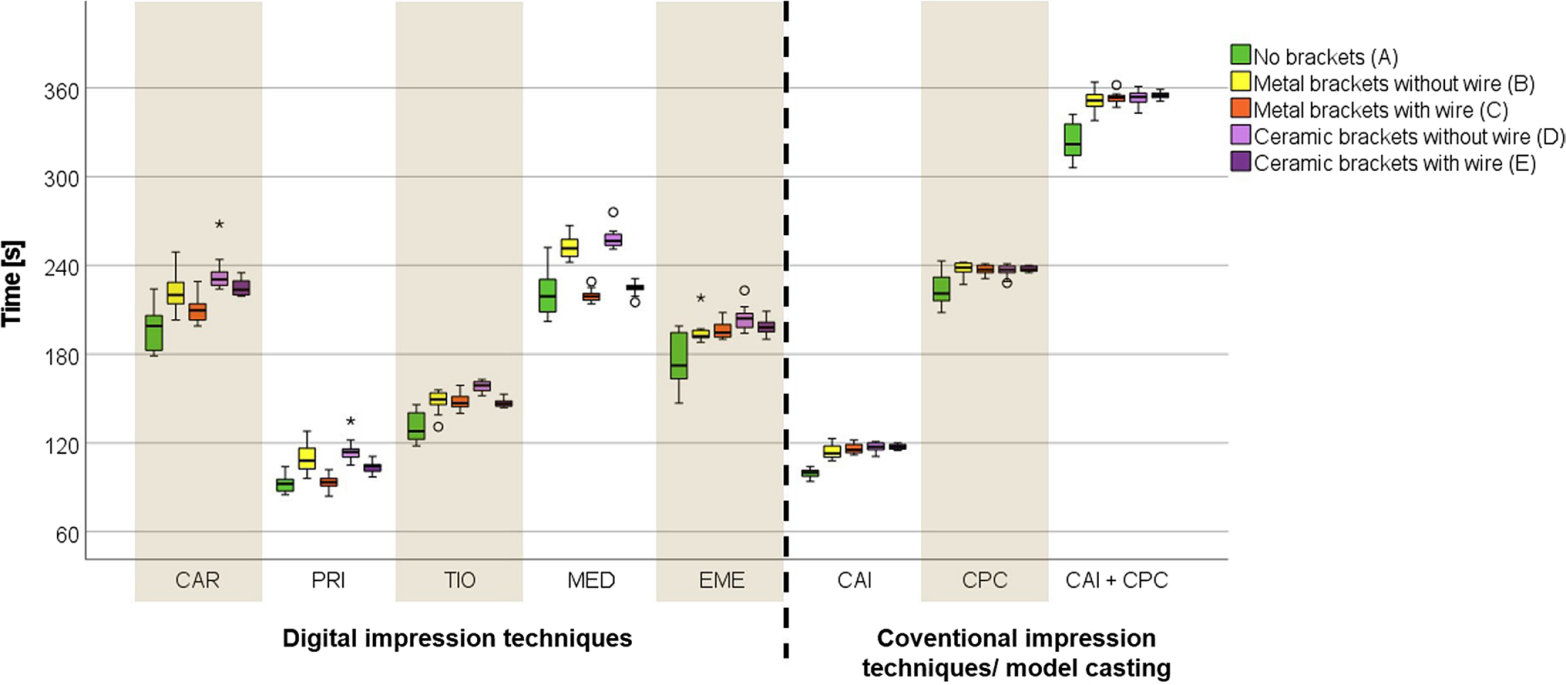
Required time[s] for impression taking in groups A–E using the different techniques (CAR = CS 3600, PRI = Primescan, TIO = TRIOS 4, MED = Medit i500, EME = Emerald S, CAI = conventional alginate impression, CPC = conventional plaster model casting, CAI + CPC = sum of conventional alginate impression and conventional plaster model casting without setting time)

Considering the required time for impression taking, the lowest amount of time was measured for the setting with no FOA (natural teeth, group A). Statistically significant differences (*p* < 0.001) were determined for each group (Table 3). On average, PRI was the fastest, followed by TIO, EME, CAR, and MED (Fig. 6; Table 3). Even though the CAI took similarly long as the intraoral scan with PRI, the CPC added almost twice the time. So overall, the combination of CAI and CPC required distinctly more time than any IOS. Almost all IOSs required more time for the groups with brackets and without wire (B and D) compared to the groups with both brackets and wire (C and E) (Fig. 6; Table 3).

**Table 3:** Required time[s] for impression taking in groups A–E reported in ascending order with respect to the mean value of the different impression techniques (PRI = Primescan, CAI = conventional alginate impression, TIO = TRIOS 4, EME = Emerald S, CAR = CS 3600, CAI + CPC = sum of conventional alginate impression and conventional plaster model casting without setting time, MED = Medit i500); in addition, the respective *p* values of the comparison within each group (A–E) as well as the comparison within each impression technique are given

Group	Required time[s] for the different groups							*p* value
	PRI	CAI	TIO	EME	CAR	CAI + CPC	MED	
No brackets (A)	92 ± 6	100 ± 3	131 ± 10	176 ± 18	196 ± 15	224 ± 11	222 ± 16	< 0.001
Metal brackets without wire (B)	110 ± 10	114 ± 5	148 ± 7	195 ± 8	222 ± 13	237 ± 5	253 ± 8	< 0.001
Metal brackets with wire (C)	93 ± 5	116 ± 3	148 ± 5	196 ± 6	210 ± 8	237 ± 3	220 ± 4	< 0.001
Ceramic brackets without wire (D)	115 ± 8	117 ± 3	158 ± 4	204 ± 8	234 ± 12	236 ± 4	258 ± 7	< 0.001
Ceramic brackets with wire (E)	104 ± 4	117 ± 2	147 ± 3	198 ± 6	225 ± 6	238 ± 2	225 ± 5	< 0.001
Mean (A–E)	103 ± 11	113 ± 8	147 ± 11	194 ± 14	217 ± 17	234 ± 8	235 ± 19	
*p* value	< 0.001	< 0.001	< 0.001	< 0.001	< 0.001	< 0.001	< 0.001

Therefore, the second null hypothesis, that no significant differences exist between the chair-side and further processing time between the five IOSs and CAIs under natural conditions as well as with FOAs regarding the necessary amount of time, had to be rejected.

## Discussion

Although some recently published studies had investigated the performance of different IOSs in the presence of orthodontic brackets and wires, clear conclusions regarding the accuracy in terms of trueness and precision could not be drawn due to the heterogeneity of study designs and lack of reliable reference systems.

The aim of the present investigation was to perform a sound evaluation of the influence of FOAs on the transfer accuracy in terms of trueness and precision applying an established reference aid–based method [24] when using digital respectively conventional impressions. While the study design was in vitro using a phantom head, lacking clinical patient-dependent factors like saliva flow, jaw movements (unintentional or restricted), and tongue and cheek mobility as well as challenging anatomical features [36–38], it allowed the standardization of all procedures. In addition, all “clinical” and laboratory practice, model assessment, and data collection were performed by one operator. The same scan strategy was applied, because a deviation of the scan path could have a significant influence on the accuracy of full-arch impression taking [32].

Looking at the results of the present study, a comparison with data in the literature proved to be rather difficult due to the limited amount of available publications.

Starting with the general accuracy of IOSs, the measured deviations of linear distances revealed values including maximum outliers of less than 0.25 mm, which is rather low when compared to the data in literature and without clinical relevance for most orthodontic purposes. For example, two in vitro studies comparing full-arch scans of four different IOSs and either models with buccal brackets of different materials (metal/ceramic/ceramic with metal slot/resin with metal slot) with/without wire [15] or models with buccal and lingual brackets revealed deviations up to 1 mm regardless of the bracket material [15]. For lingual brackets, even higher deviations up to 2.49 mm were seen [14]. In concordance with the current results, differences between the tested IOSs were observed. To determine accuracy, in those studies the distance between the canines and the molars was digitally measured for IOSs and compared to manually measured values of the conventional study models [14, 15]. This method might be a possible reason for the relatively large deviations. Although diagnostic measurements on plaster casts have been undertaken manually in orthodontics for decades, the reference value for accuracy of IOSs in terms of trueness and precision obtained by manual measurements has to be interpreted with care, as the exactness of manual measurements in comparison to digital measurements is questionable. Furthermore, no information was given about the number of individuals performing the manual measurements as well as the intra- and interobserver reliability [14, 15]. Even if the IOS Trios, which performed well in those studies [14, 15], was also used in the present study (TIO), it has to be considered that both the hardware and software components have been enhanced since then, hampering the comparability of the respective data [25].

When it comes to the influence of different bracket materials (metal/ceramic/resin) on the performance of different IOSs, it was interesting to see that some IOSs revealed substantially lower deviations regarding the measured distances in the groups with FOAs than in the group without. This is in contrast to the findings of an in vitro study by Song and Kim [16], who determined the lowest deviation for the model without FOA. They also compared scans of models with and without different bracket materials (metal/ceramic/resin); in contrast to the present study, the scans were performed in the presence of artificial saliva [16]. Reference data were obtained by a laboratory scan of the models without artificial saliva; the accuracy was determined by a best-fit superimposition of the IOSs and the reference data. Two of the respective IOSs were also used in the present study (CAR, MED) as well as the Trios 3, a predecessor model of the current IOS TIO. Song and Kim [16] did not show significant differences within the IOSs, which is in contrast to the findings of the present study. However, they found significant differences between the different bracket models: resin and metal brackets showed larger deviations than ceramic brackets. The present investigation also revealed larger deviations in metallic than in ceramic brackets. In comparison to the present results, the deviations measured by Song and Kim [16] were considerably larger, which could be due to the presence of artificial saliva in the reference study. The use of artificial saliva in in vitro studies should be considered thoroughly: on the one hand, the in vitro situation can become closer to an in vivo approach, while on the other hand, limitations of artificial saliva might occur as unequal application or difference in composition when compared to individual patient saliva. Furthermore, all manufacturers of IOSs recommend the drying of tooth surfaces with oil-free air before scanning. Therefore, no artificial saliva was used in the present study design.

Another in vitro study claimed to assess the accuracy of one predecessor model of the IOS TIO using plaster casts with metal/ceramic brackets in comparison to plaster casts without brackets [18]. They found significant differences between the models without brackets and those with both kinds of brackets and reported the highest deviations for ceramic brackets—which is different when compared to all other corresponding data in the literature. In addition, the data regarding accuracy have to be interpreted with caution, as on the one hand plaster casts have a different refractive index compared to plastic model teeth used in other studies [14–16] respectively natural teeth used in the present study. On the other hand, a best-fit superimposition approach was used with subsequent measurements of distances in cross-sections of canines and molars [18]. Therefore, the measured distances display only deviations between the superimposed datasets and not the accuracy of the scanning method, as the measured deviations in best-fit superimpositions are the summarized deviations of the scanning method plus the superimposition method. A proper 3D evaluation might have generated different results.

Moreover, two recent studies compared full-arch scans before and after the insertion of metal brackets without wires in vivo [17, 20]. Kang et al. [20] used a best-fit superimposition with subsequent measurements of intercanine and intermolar distances and found neither significant differences between the two scanners used nor between the situation before and after bracket bonding. In concordance to the present study, the shorter intercanine distance (comparable to D2_3) showed less deviation compared to the long-span intermolar distance (comparable to D1_4) [20]. Interestingly, the mean deviations observed by Kang et al. [20] for the intercanine distance were within the range of deviations seen in the present study, while the deviations for the intermolar distance were substantially larger. Another in vivo approach compared a best-fit superimposition of two full-arch scans prior to orthodontic bracket bonding with another scan after insertion of metal brackets without wires using an IOS also investigated in the present study (CAR) [17]. A significant increase of deviations after bracket bonding in all regions (anterior, premolars, molars) was observed which was in the upper range of deviations recorded in the present study for the comparable setting (CAR, group B).

Altogether, besides the large variety of study designs and methods described in the current literature, contradictory results regarding the influence of FOAs in IOSs exist. Furthermore, even though all recent studies refer to the term “accuracy” and some also consider trueness and precision, different notions in the context of those terms impede their interpretation [14–18, 20]. Therefore, the current study is the first one using a standardized method according to ISO 5725–1 [35] to determine the transfer accuracy in terms of trueness and precision of IOSs in presence of FOAs.

Regarding the required time for conventional and digital impressions, a longer scanning time for models with FOAs compared to models with natural teeth was determined in the present investigation, whereas the difference between ceramic and metal brackets was rather small. All in all, CAI and PRI were significantly faster than the other tested IOSs (range: 102.7 ± 11.14 to 112.92 ± 7.60 vs. 146.63 ± 10.73 to 235.32 ± 18.79). Besides the present study, to our knowledge only one other in vitro study dealt with the required time for intraoral scans in models with FOAs and showed a significantly (*p* < 0.001) shorter scanning time for models without brackets (48.87 ± 7.26) compared to models with metal (102.17 ± 10.61) and ceramic (234.10 ± 34.98) brackets, whereby scans of ceramic brackets took on average more than 3 min longer than without any FOA [18].

Most studies in the literature dealing with orthodontic patients investigated digital and conventional (alginate) full-arch impressions without any fixed appliances [10–13, 27] and found the chair-side time to be equally [11, 12] respectively significantly shorter for alginate impressions [27]. Nevertheless, the present results are in concordance with Grünheid et al. [27] due to the fact that the subsequently needed laboratory time for CAI equals respectively extends the chair-side time benefits of CAI compared to IOSs. Furthermore, the time needed for IOSs could depend on the processing power of the respective hard- and software, which to date cannot be verified due to the lack of comparable studies using different hard- and software versions. Interestingly, the IOSs CAR, PRI, TIO, and MED needed up to 16% less time for scans of metal and ceramic brackets with wire than without. Perhaps the inserted wire acts as guidance tool for the scanning software which allows a faster processing of the scanned images. Due to the fact that the accuracy of those IOS (especially CAR, PRI, and TIO) was comparable with and without wire, whereas the accuracy of CAIs decreased substantially in the groups with wire, the use of the respective IOSs can be clinically recommended in terms of both accuracy and time efficiency because a removal of the wire prior to scanning seems not to be necessary.

It can be considered a limitation that only the occlusal and no buccal or lingual surfaces were considered for measurements. The reason for this restriction was the objective of using a defined and stable reference structure to compare the measurements to. The advantage of this system respectively procedure was the fact that it had been proved to be reliable in other investigations before. So far—unfortunately—no such reference structure has been described for buccal or lingual tooth surfaces.

Nevertheless, to verify the present results, a re-investigation in a clinical setting should be performed—particularly regarding the influence of saliva. Furthermore, disinfection of alginate impressions is required in patient care to prevent a contamination transmission from the dental chair to the laboratory. However, due to the water-based character, alginates might swell during disinfection what might have an impact on accuracy [33]. As the level of digitalization in the dental area will proceed, digital impressions will increase and most probably replace CAIs long term, which seems to be especially beneficial in the presence of FOAs.

## Conclusion

Fixed orthodontic appliances in terms of buccal brackets without/with wire influence the transfer accuracy of full-arch impressions, irrespective of the bracket material (metal/ceramic). The use of digital intraoral scans shows a positive effect when compared with conventional alginate impressions; the use of CAIs was more prone to disturbances caused by the presence of FOAs and in total more time-consuming than the use of IOSs—even if CAI showed the smallest average deviations in settings without FOAs. Thus, within the limitations of this in vitro study, IOSs can be recommended for impression taking with FOA. To verify the present results and overcome the limitations of an in vitro setting, a re-investigation in a clinical setting should be performed.

## Supplementary Information

Below is the link to the electronic supplementary material.

Supplementary file1 (PDF 120 KB)
