# Supplementary material for: The transfer accuracy of digital and conventional full-arch impressions influenced by fixed orthodontic appliances: a reference aid–based in vitro study
Source: Clin Oral Investig. 2022 Sep 15;27(1):273–83. doi: 10.1007/s00784-022-04721-5 (PMC9876864; doi:10.1007/s00784-022-04721-5)
Supplement: Supplementary file 1 — Supplementary file1 (PDF 120 KB) [file 784_2022_4721_MOESM1_ESM.pdf]

Suppl. Table 1. Data of the linear distances (D1_2, D1_3, D1_4, D2_3, D2_4, D3_4) for the different groups A-E reported for each different impression technique (CAR=CS 3600, PRI=Primescan, TIO=Trios 4, MED=Medit i500, EME=Emerald S, CAI=conventional alginate impression) as mean for trueness [µm] and standard deviation (SD) for precision [µm] according to the International Organization for Standardization (ISO) 5725-135. In addition, the respective p-values of the comparison within each group are given.

**Suppl. Table 1**		**Group**									
		**No brackets (A)**		**Metal brackets without wire (B)**		**Metal brackets with wire (C)**		**Ceramic brackets without wire (D)**		**Ceramic brackets with wire (E)**	
**Linear distance**	**Impression technique**	Mean [µm] ± SD [µm]	p	Mean [µm] ± SD [µm]	p	Mean [µm] ± SD [µm]	p	Mean [µm] ± SD [µm]	p	Mean [µm] ± SD [µm]	p
D1_2	CAR	65 ± 11	<0.001	59 ± 19	<0.001	49 ± 17	<0.001	54 ± 10	<0.001	62 ± 15	<0.001
	PRI	18 ± 11		35 ± 9		21 ± 7		7 ± 3		36 ± 10	
	TIO	11 ± 11		16 ± 13		12 ± 12		6 ± 5		3 ± 2	
	MED	28 ± 15		20 ± 11		20 ± 10		24 ± 9		35 ± 9	
	EME	11 ± 6		12 ± 12		11 ± 13		6 ± 6		4 ± 3	
	CAI	15 ± 11		101 ± 82		136 ± 108		152 ± 79		174 ± 109	
D1_3	CAR	67 ± 47	0.102	45 ± 32	0.014	49 ± 31	<0.001	64 ± 43	<0.001	27 ± 25	<0.001
	PRI	40 ± 34		35 ± 20		40 ± 35		40 ± 31		36 ±35	
	TIO	55 ± 50		37 ± 32		44 ± 41		51 ± 37		44 ± 31	
	MED	84 ± 53		108 ± 60		179 ± 101		109 ± 68		154 ± 75	
	EME	49 ± 39		34 ± 32		47 ± 39		58 ± 34		37 ± 34	
	CAI	39 ± 18		82 ± 79		226 ± 201		182 ± 67		231 ± 295	
D1_4	CAR	82 ± 57	<0.001	112 ± 82	0.013	61 ± 43	<0.001	136 ± 65	0.012	55 ± 46	<0.001
	PRI	45 ± 26		104 ± 68		75 ± 54		64 ± 49		84 ± 68	
	TIO	89 ± 67		71 ± 81		69 ± 36		84 ± 58		146 ± 86	
	MED	186 ± 111		214 ± 118		314 ± 177		191 ± 115		269 ± 136	
	EME	93 ± 46		60 ± 55		128 ± 73		97 ± 60		93 ± 60	
	CAI	31 ± 22		121 ± 142		212 ± 204		104 ± 81		141 ± 153	
D2_3	CAR	39 ± 22	<0.001	31 ± 19	0.002	25 ± 18	<0.001	24 ± 15	<0.001	30 ± 23	<0.001
	PRI	26 ± 19		32 ± 18		29 ± 21		43 ± 25		32 ± 23	
	TIO	33± 25		29 ± 19		27 ± 26		34 ± 24		36 ± 18	
	MED	72 ± 43		94 ± 53		140 ± 69		71 ± 43		101 ± 56	
	EME	29 ± 33		57 ± 30		26 ± 25		48 ± 35		25 ± 17	
	CAI	15 ± 26		120 ± 103		295 ± 118		186 ± 101		341 ± 169	
D2_4	CAR	42 ± 29	<0.001	68 ± 49	0.005	63 ± 36	<0.001	38 ± 37	0.002	88 ± 39	<0.001
	PRI	22 ± 16		60 ± 36		48 ± 29		46 ± 27		45 ± 39	
	TIO	55 ± 27		37 ± 46		48 ± 22		56 ± 37		105 ± 62	
	MED	110 ± 60		136 ± 65		179 ± 102		84 ± 58		110 ± 79	
	EME	33 ± 38		65 ± 39		56 ± 35		41 ± 26		33 ± 33	
	CAI	14 ± 13		145 ± 130		257 ± 168		166 ± 112		313 ± 237	
D3_4	CAR	91 ± 13	<0.001	70 ± 19	<0.001	66 ± 19	<0.001	72 ± 15	<0.001	78 ± 14	<0.001
	PRI	20 ± 13		37 ± 13		19 ± 16		6 ± 5		36 ± 8	
	TIO	21 ± 13		14 ± 8		15 ± 10		15 ± 7		22 ± 10	
	MED	24 ± 18		21 ± 11		14 ± 13		25 ± 9		35 ± 12	
	EME	13 ± 8		13 ± 15		13 ± 6		6 ± 5		9 ± 6	
	CAI	14 ± 15		50 ± 45		87 ± 52		67 ± 80		71 ± 53	

Suppl. Table 2. P-values of the pairwise comparison of the different impression techniques (CAR=CS 3600, PRI=Primescan, TIO=Trios 4, MED=Medit i500, EME=Emerald S, CAI=conventional alginate impression) in terms of linear distances (D1_2, D1_3, D1_4, D2_3, D2_4, D3_4) for the different groups A-E as mean for trueness [µm] and standard deviation (SD) for precision [µm] according to the International Organization for Standardization (ISO) 5725-135.

Suppl. Table 2		Group																								
		No brackets (A)					Metal brackets without wire (B)					Metal brackets with wire (C)					Ceramic brackets without wire (D)					Ceramic brackets with wire (E)				
Linear distance	Impression technique	CAR	EME	MED	TIO	PRI	CAR	EME	MED	TIO	PRI	CAR	EME	MED	TIO	PRI	CAR	EME	MED	TIO	PRI	CAR	EME	MED	TIO	PRI
D1_2	EME	<0.001	-	-	-	-	<0.001	-	-	-	-	<0.001	-	-	-	-	<0.001	-	-	-	-	<0.001	-	-	-	-
	MED	0.012	0.014	-	-	-	<0.001	0.272	-	-	-	0.006	0.138	-	-	-	0.110	0.006	-	-	-	0.042	0.005	-	-	-
	TIO	<0.001	0.804	0.007	-	-	<0.001	0.532	0.636	-	-	<0.001	0.826	0.206	-	-	<0.001	0.961	0.007	-	-	<0.001	0.830	0.002	-	-
	PRI	<0.001	0.272	0.177	0.178	-	0.091	0.004	0.069	0.022	-	0.008	0.117	0.934	0.178	-	<0.001	0.602	0.025	0.636	-	0.055	0.003	0.911	0.002	-
	CAI	<0.001	0.520	0.071	0.372	0.650	0.965	<0.001	0.001	<0.001	0.099	0.369	<0.001	<0.001	<0.001	<0.001	0.172	<0.001	0.003	<0.001	<0.001	0.337	<0.001	0.003	<0.001	0.004
D1_3	EME	0.228	-	-	-	-	0.344	-	-	-	-	0.849	-	-	-	-	0.838	-	-	-	-	0.615	-	-	-	-
	MED	0.453	0.050	-	-	-	0.024	0.001	-	-	-	0.004	0.002	-	-	-	0.160	0.108	-	-	-	<0.001	0.001	-	-	-
	TIO	0.352	0.785	0.092	-	-	0.479	0.811	0.003	-	-	0.633	0.774	0.001	-	-	0.592	0.740	0.052	-	-	0.342	0.654	0.003	-	-
	PRI	0.082	0.592	0.013	0.418	-	0.668	0.605	0.007	0.781	-	0.578	0.715	0.001	0.938	-	0.195	0.275	0.007	0.447	-	0.815	0.789	<0.001	0.473	-
	CAI	0.134	0.770	0.025	0.572	0.807	0.447	0.088	0.134	0.142	0.234	0.004	0.002	0.957	0.001	0.001	0.001	0.001	0.071	<0.001	<0.001	0.001	0.007	0.453	0.024	0.003
D1_4	EME	0.565	-	-	-	-	0.119	-	-	-	-	0.036	-	-	-	-	0.188	-	-	-	-	0.186	-	-	-	-
	MED	0.026	0.098	-	-	-	0.075	0.001	-	-	-	<0.001	0.052	-	-	-	0.447	0.038	-	-	-	<0.001	0.002	-	-	-
	TIO	0.903	0.650	0.035	-	-	0.215	0.748	0.003	-	-	0.740	0.077	<0.001	-	-	0.052	0.532	0.007	-	-	0.011	0.226	0.058	-	-
	PRI	0.195	0.061	<0.001	0.156	-	0.934	0.139	0.062	0.248	-	0.619	0.110	<0.001	0.868	-	0.014	0.258	0.001	0.612	-	0.377	0.661	<0.001	0.099	-
	CAI	0.036	0.008	<0.001	0.026	0.424	0.424	0.447	0.010	0.661	0.473	0.011	0.657	0.133	0.027	0.041	0.188	1.000	0.038	0.532	0.258	0.070	0.622	0.009	0.473	0.352
D2_3	EME	0.130	-	-	-	-	0.058	-	-	-	-	0.872	-	-	-	-	0.131	-	-	-	-	0.729	-	-	-	-
	MED	0.142	0.003	-	-	-	0.005	0.354	-	-	-	<0.001	<0.001	-	-	-	0.007	0.244	-	-	-	0.006	0.002	-	-	-
	TIO	0.523	0.383	0.035	-	-	0.992	0.056	0.005	-	-	0.969	0.841	<0.001	-	-	0.523	0.415	0.048	-	-	0.523	0.325	0.036	-	-
	PRI	0.186	0.849	0.005	0.495	-	0.930	0.070	0.006	0.922	-	0.804	0.682	0.001	0.834	-	0.129	0.996	0.246	0.413	-	0.800	0.549	0.013	0.700	-
	CAI	0.003	0.137	<0.001	0.018	0.093	0.009	0.479	0.826	0.009	0.012	<0.001	<0.001	0.254	<0.001	<0.001	<0.001	0.002	0.048	0.486	0.002	<0.001	<0.001	0.062	<0.001	<0.001
D2_4	EME	0.266	-	-	-	-	0.981	-	-	-	-	0.762	-	-	-	-	0.582	-	-	-	-	0.016	-	-	-	-
	MED	0.005	<0.001	-	-	-	0.027	0.029	-	-	-	0.005	0.002	-	-	-	0.031	0.110	-	-	-	0.830	0.009	-	-	-
	TIO	0.277	0.028	0.088	-	-	0.112	0.106	<0.001	-	-	0.410	0.602	<0.001	-	-	0.163	0.399	0.450	-	-	0.736	0.006	0.903	-	-
	PRI	0.100	0.595	<0.001	0.006	-	0.834	0.815	0.016	0.168	-	0.377	0.562	<0.001	0.953	-	0.393	0.762	0.194	0.588	-	0.055	0.629	0.033	0.024	-
	CAI	0.011	0.156	<0.001	<0.001	0.375	0.226	0.236	0.320	0.005	0.156	<0.001	<0.001	0.418	<0.001	<0.001	<0.001	0.001	0.099	0.016	0.003	0.110	<0.001	0.166	0.207	<0.001
D3_4	EME	<0.001	-	-	-	-	<0.001	-	-	-	-	<0.001	-	-	-	-	<0.001	-	-	-	-	<0.001	-	-	-	-
	MED	0.001	0.122	-	-	-	<0.001	0.138	-	-	-	<0.001	0.781	-	-	-	0.017	0.001	-	-	-	0.004	0.001	-	-	-
	TIO	<0.001	0.153	0.907	-	-	<0.001	0.595	0.341	-	-	<0.001	0.792	0.588	-	-	<0.001	0.091	0.132	-	-	<0.001	0.086	0.132	-	-
	PRI	<0.001	0.254	0.685	0.773	-	0.062	0.001	0.087	0.008	-	<0.001	0.633	0.450	0.830	-	<0.001	0.819	0.001	0.055	-	0.013	<0.001	0.718	0.062	-
	CAI	<0.001	0.926	0.101	0.128	0.217	0.031	0.004	0.156	0.018	0.770	0.895	<0.001	<0.001	<0.001	0.001	0.208	<0.001	0.256	0.008	<0.001	0.093	<0.001	0.242	0.007	0.418
